# Dietary intake of amino acids and vitamins compared to NRC requirements in obese cats undergoing energy restriction for weight loss

**DOI:** 10.1186/s12917-020-02649-0

**Published:** 2020-11-07

**Authors:** Caitlin E. Grant, Anna K. Shoveller, Shauna Blois, Marica Bakovic, Gabrielle Monteith, Adronie Verbrugghe

**Affiliations:** 1 Department of Clinical Studies, Ontario Veterinary College, University of Guelph, Guelph, ON N1G 2W1 Canada; 2 Department of Animal Biosciences, Ontario Agricultural College, University of Guelph, Guelph, ON N1G 2W1 Canada; 3 Department of Human Health & Nutritional Science, College of Biological Sciences, University of Guelph, Guelph, ON N1G 2W1 Canada

**Keywords:** Energy restriction, Feline obesity, Essential nutrients, Choline, Arginine

## Abstract

**Background:**

This study aimed to determine if obese cats undergoing energy restriction for weight loss would meet the National Research Council’s (NRC) indispensable amino acid and vitamin recommendations when fed a purpose-formulated diet. Thirty cats were placed into one of two groups; obese (BCS 8 to 9/9; *n* = 16) and lean (BCS 4 to 5/9; *n* = 14) and included in a non-randomized retrospective observational study. Cats were fed a veterinary weight loss food during a 4-week period of weight maintenance. Obese cats (O-MAINT) refers to obese cats during this period, L-MAINT to lean cats. After this initial 4-week period, the lean cats finished the study at this time and the 16 obese cats continued and were energy restricted for a 10-week period (O-RESTRICT). Analysis for dietary concentrations of indispensable amino acid and vitamin contents were performed. Daily food intakes were used to determine minimum, maximum and average daily intakes of individual nutrients for all three groups and compared against NRC 2006 minimum requirements (MR), adequate intakes (AI) and recommended allowances (RA) for adult cats.

**Results:**

Over 10 weeks, O-RESTRICT cats lost 672 g ± 303 g, representing a weight loss rate of 0.94 ± 0.28% per week. Daily intake of the majority of indispensable amino acids and vitamins was greater than the NRC 2006 recommended allowance (RA per kg ideal body weight ^0.67), except for arginine, choline, crude protein, phenylalanine plus tyrosine and threonine. All O-RESTRICT cats had minimum, average, and maximum arginine intakes less than the NRC AI. Minimum daily intake of choline was below NRC RA for all O-RESTRICT cats and below NRC MR for two. All, except one, O-RESTRICT cats had a maximum and average choline intake below RA.

**Conclusions:**

All cats remained clinically healthy and showed no clinical signs of deficiency. Dietary choline and arginine requirements of obese cats as well as health risks associated with low dietary intake during energy restriction warrant further investigation.

## Background

Obesity is defined as excess body fat accumulation that can have negative impacts on overall health [1] and has been reported to be the second most common health problem in the domestic cat population in developed countries [2]. Obesity is the result of a positive energy balance where the energy consumed is greater than the energy expended. Energy restriction is paramount to successful weight loss, but the amount of restriction required to lose weight can vary between individuals and some require severe calorie restriction [3]. The goal of weight loss is to restrict the provision of dietary energy without nutrient restriction as the majority of nutrients are primarily required on a body weight basis. When calories are restricted, intake of essential nutrients including indispensable amino acids, essential fatty acids, vitamins and minerals may also be less, even though they are supplemented in purpose formulated weight loss diets. Previous research in obese dogs found the intake of selenium, choline, methionine and cysteine, tryptophan, total fat, magnesium and potassium to be lower than recommended allowances according to the National Research Council (NRC) when dogs were put on a weight loss plan with a veterinary therapeutic weight loss food [4, 5]. This research has been done only in dogs and information is lacking on the provision of adequate amounts of essential nutrients during energy restriction in obese cats. The fact that there is evidence suggesting intake of some essential nutrients below NRC recommendations for canine diets though leads us to believe the same is likely true of feline diets. The objective of the current study was to investigate dietary intake of indispensable amino acids and vitamins in lean cats fed to maintain body weight, obese cats fed to maintain body weight, and obese cats undergoing dietary energy restriction for weight loss utilizing a veterinary therapeutic weight loss food and compare vitamin and amino acid intake with NRC recommendations.

## Results

### Energy intake and weight loss

All cats tolerated the diet, remained clinically healthy for the duration of the study and demonstrated no clinical signs related to any nutrient deficiency. Energy intake and weight loss were reported previously [6]. In summary average daily energy intake for L-MAINT group was 272.3 (+/− 46.5) kcals per day. Average daily energy intake for O-MAINT group was 221.5 (+/− 24.4) kcals per day and for O-RESTRICT this restricted to 138.2 (+/− 10.2) kcals per day. Obese cats lost a total average of 672 (+/− 303) grams over the 10-week period which is an average weight loss rate of 0.94 (+/− 0.28) % of initial body weight per week.

### Biochemistry data

Serum glucose, cholesterol and TG are reported in Table 1. Both serum glucose and cholesterol were within the reference ranges for all 3 groups and did not change with energy restriction. Serum TG was higher in O-MAINT compared to L-MAINT (*P* = 0.03), but not different between those groups and O-RESTRICT.

**Table 1:** Biochemical parameters from 14 lean (L-MAINT) and 16 obese cats during maintenance (O-MAINT) and during energy restriction (O-RESTRICT)

Parameter	L-MAINT	O-MAINT	O-RESTRICT	Reference Range
Glucose (mmol/L)	4.8 (3.0–7.8)	4.8000 (3.4–9.9)	4.95 (3.9–10.2)	4.4–7.7
Cholesterol (mmol/L)	7.12 ± 4.6	6.83 ± 3.95	6.75 ± 14.62	2–12
TG (mmol/L)	9.0 (9.4–19.8) ^A^	11.7 (7.2–86.4) ^B^	9.9 (7.2–43.2) ^AB^	–

Normally distributed data expressed as mean ± SE

Non-parametric data expressed as median (min-max)

Superscript capital letters (A, B) denote significant differences between groups with different letters where a *p*-value < 0.05 is considered significant

No superscript letters in a row indicates no significant differences between groups for the measured parameter

### Dietary crude protein, amino acid and vitamin intake

Dietary intake (grams per kg BW^0.67^) of crude protein, and each amino acid and vitamin differed between groups (L-MAINT, O-MAINT, O-RESTRICT). Minimum intake of each nutrient differed among groups with the O-MAINT group having the greatest mean minimum nutrient intake and the O-RESTRICT having the least, while L-MAINT was intermediate. When a Tukey-Kramer adjustment was applied to account for multiple comparisons, only the difference between O-MAINT and O-RESTRICT (*P* = 0.0001) and between L-MAINT and O-MAINT (*P* = 0.04) remained significant. Differences between groups were also noted for maximum intake of each nutrient, with again O-MAINT having the greatest mean maximum intake (grams per kg BW^0.67^) and O-RESTRICT having the least, while L-MAINT was intermediate. When a Tukey-Kramer adjustment was applied, all differences between groups remained significant (*P* ≤ 0.001 for all comparisons). For the average intake of each nutrient, no differences were noted between L-MAINT and O-MAINT. However, the differences between the L-MAINT and O-RESTRICT (*P* ≤ 0.001) as well as between O-MAINT and O-RESTRICT (*P* ≤ 0.001) were significant with the O-RESTRICT average being the lowest, O-MAINT the highest and L-MAINT intermediate. When a Tukey-Kramer adjustment was applied, the differences remained significant.

### Comparison with NRC 2006 recommendations – crude protein and amino acids

Minimum, average and maximum dietary intakes of crude protein were above NRC MR in all cats in the O-MAINT and O-RESTRICT groups (Table 2). One cat in the O-MAINT group had a minimum crude protein intake below NRC RA (Table 3). Also, one cat in the O-RESTRICT group had a minimum, average and maximum crude protein intake that was less than NRC RA (Table 3). Dietary intakes for most amino acids were greater than the NRC recommendations for all cats (Table 2). One cat also had a minimum phenylalanine + tyrosine intake that was less than NRC AI (Table 3). Though the most significant findings were for arginine. Four cats in the O-MAINT group had minimum arginine intakes less than the NRC AI and one of these cats also had an average arginine intake less than the NRC AI (Table 3). All 16 cats in the O-RESTRICT group had minimum, average, and maximum arginine intakes less than the NRC AI (Table 3).

**Table 2:** Daily intake of crude protein and indispensable amino acids in 14 lean cats during maintenance (L-MAINT) and in 16 obese cats during maintenance (O-MAINT) and during energy restriction (O-RESTRICT), compared to NRC minimum requirement, adequate intake and recommended allowance

Nutrient	NRC^g^			Dietary intake		
	NRC MR^d^	NRC AI^e^	NRC RA^f^	L-MAINT	O-MAINT	O-RESTRICT
**Minimum**^**a**^						
Crude Protein (g)	3.97	–	4.96	6.39 (3.92–10.78)	9.19 (4.60–10.68)	5.75 (4.94–6.35)
Arginine (g)	–	0.19	–	0.17 (0.10–0.28)	0.24 (0.12–0.28)	0.15 (0.13–0.17)
Histidine (g)	–	0.064	–	0.11 (0.07–0.19)	0.16 (0.08–0.19)	0.10 (0.09–0.11)
Isoleucine (g)	–	0.11	–	0.21 (0.13–0.36)	0.31 (0.15–0.36)	0.19 (0.16–0.21)
Leucine (g)	–	0.25	–	0.60 (0.37–1.01)	0.86 (0.43–1.00)	0.54 (0.46–0.59)
Lysine (g)	–	0.084	–	0.26 (0.16–0.45)	0.38 (0.19–0.45)	0.24 (0.21–0.27)
Methionine (g)	0.033	–	0.042	0.49 (0.30–0.83)	0.71 (0.35–0.82)	0.44 (0.38–0.49)
Methionine + Cysteine (g)	0.067	–	0.084	0.68 (0.42–1.15)	0.98 (0.49–1.14)	0.61 (0.53–0.68)
Phenylalanine (g)	–	0.099	–	0.28 (0.17–0.48)	0.41 (0.20–0.48)	0.26 (0.22–0.28)
Phenylalanine + Tyrosine (g)	–	0.38	–	0.51 (0.31–0.86)	0.74 (0.37–0.86)	0.46 (0.40–0.51)
Threonine (g)	–	0.13	–	0.19 (0.12–0.33)	0.28 (0.14–0.32)	0.17 (0.15–0.19)
Tryptophan (g)	–	0.032	0.032	0.22 (0.14–0.37)	0.32 (0.16–0.37)	0.20 (0.18–0.22)
Valine (g)	–	0.13	–	0.27 (0.17–0.46)	0.39 (0.2–046)	0.25 (0.21–0.27)
Taurine (g)	0.0079	–	0.0099	0.08 (0.05–0.13)	0.11 (0.05–0.13)	0.07 (0.06–0.08)
**Maximum**^**b**^						
Crude Protein (g)	3.97	–	4.96	10.76 (8.96–10.96)	9.50 (8.52–10.68)	5.89 (4.94–6.58)
Arginine (g)	–	0.19	–	0.28 (0.23–0.29)	0.25 (0.22–0.28)	0.15 (0.13–0.17)
Histidine (g)	–	0.064	–	0.19 (0.16–0.20)	0.17 (0.15–0.19)	0.11 (0.09–0.12)
Isoleucine (g)	–	0.11	–	0.36 (0.30–0.37)	0.32 (0.28–0.36)	0.20 (0.16–0.22)
Leucine (g)	–	0.25	–	1.00 (0.84–1.02)	0.89 (0.79–1.00)	0.55 (0.46–0.61)
Lysine (g)	–	0.084	–	0.45 (0.37–0.45)	0.40 (0.36–0.45)	0.25 (0.21–0.28)
Methionine (g)	0.033	–	0.042	0.83 (0.69–0.84)	0.73 (0.66–0.82)	0.45 (0.38–0.51)
Methionine + Cysteine (g)	0.067	–	0.084	1.15 (0.95–1.17)	1.01 (0.91–1.14)	0.63 (0.53–0.70)
Phenylalanine (g)	–	0.099	–	0.48 (0.40–0.49)	0.42 (0.38–0.48)	0.26 (0.22–0.29)
Phenylalanine + Tyrosine (g)	–	0.38	–	0.86 (0.72–0.88)	0.76 (0.68–0.86)	0.47 (0.40–0.53)
Threonine (g)	–	0.13	–	0.32 (0.27–0.33)	0.29 (0.26–0.32)	0.18 (0.15–0.20)
Tryptophan (g)	–	0.032	0.032	0.37 (0.31–0.38)	0.33 (0.29–0.37)	0.21 (0.19–0.23)
Valine (g)	–	0.13	–	0.46 (0.38–0.47)	0.41 (0.37–0.46)	0.25 (0.21–0.28)
Taurine (g)	0.0079	–	0.0099	0.13 (0.11–0.13)	0.11 (0.10–0.13)	0.07 (0.06–0.08)
**Average**^**c**^						
Crude Protein (g)	3.97	–	4.96	9.06 (6.55–10.80)	9.32 (6.60–10.68)	5.83 (4.94–6.58)
Arginine (g)	–	0.19	–	0.23 (0.17–0.28)	0.24 (0.17–0.28)	0.15 (0.13–0.17)
Histidine (g)	–	0.064	–	0.16 (0.12–0.19)	0.17 (0.12–0.19)	0.10 (0.09–0.12)
Isoleucine (g)	–	0.11	–	0.30 (0.22–0.36)	0.31 (0.22–0.36)	0.19 (0.16–0.22)
Leucine (g)	–	0.25	–	0.83 (0.61–1.01)	0.87 (0.62–1.00)	0.54 (0.46–0.61)
Lysine (g)	–	0.084	–	0.37 (0.27–0.45)	0.39 (0.28–0.45)	0.24 (0.21–0.27)
Methionine (g)	0.033	–	0.042	0.68 (0.50–0.83)	0.72 (0.51–0.82)	0.45 (0.38–0.51)
Methionine + Cysteine (g)	0.067	–	0.084	0.94 (0.70–1.15)	1.00 (0.70–1.14)	0.62 (0.53–0.70)
Phenylalanine (g)	–	0.099	–	0.39 (0.29–0.48)	0.41 (0.29–0.48)	0.26 (0.22–0.29)
Phenylalanine + Tyrosine (g)	–	0.38	–	0.71 (0.53–0.87)	0.75 (0.53–0.86)	0.47 (0.40–0.53)
Threonine (g)	–	0.13	–	0.27 (0.20–0.33)	0.28 (0.20–0.32)	0.18 (0.15–0.20)
Tryptophan (g)	–	0.032	0.032	0.31 (0.23–0.37)	0.32 (0.23–0.37)	0.20 (0.18–0.23)
Valine (g)	–	0.13	–	0.38 (0.28–0.46)	0.4 (0.28–0.46)	0.25 (0.21–0.28)
Taurine (g)	0.0079	–	0.0099	0.11 (0.08–0.13)	0.11 (0.08–0.13)	0.07 (0.06–0.08)

The daily intake of all essential nutrients was calculated from each cat’s food intake and the average nutrient content of the diet (Table 5) and expressed per kg BW ^0.67. Results are written as median (range)

^a^Minimum daily intake defined as the least daily intake that the cat consumed during the maintenance period

^b^Maximum daily intake defined as the greatest daily intake that the cat consumed during the maintenance period

^c^Average daily intake was defined as the mean daily intake for the whole maintenance period

^d^ Minimum requirement: the minimal concentration of a maximally bioavailable nutrient that will support a defined physiological state [4]

^e^ Adequate intake: the concentration of nutrient demonstrated to support a defined physiological state when no minimal requirement has been demonstrated [4]

^f^ Recommended allowance: the concentration of nutrient demonstrated to support a defined physiological stage [4]

^g^ All requirements are expressed as the unite stated per kg BW^0.67

**Table 3:** Daily intake of essential nutrients in 14 lean cats during maintenance (L-MAINT) and in 16 obese cats during maintenance (O-MAINT) and during energy restriction (O-RESTRICT), compared to NRC minimum requirement, adequate intake and recommended allowance. The frequency and prevalence of not meeting NRC requirement for essential nutrient intake for nutrients for which at least one cat in the group was below the requirement as well as the mean difference between essential nutrient intake and NRC requirement

Group	Period	Nutrient	Intake	NRC MR^g^				NRC AI^h^				NRC RA^i^			
				Req.^d^	No. Less^e^	Diff.^f^	*p*-value	Req.	No. Less	Diff.	*p*-value	Req.	No. Less	Diff.	*p*-value
**L-MAINT**	**Min**^**a**^	CP (g)	6.39 (3.92–10.78)	3.97	1	−2.92	0.0005					4.96	4	−1.93	0.0096
		Arg (mg)	0.17 (0.10–0.28)					0.19	9	0.01	0.6022				
		Phe + Tyr (mg)	0.51 (0.31–0.86)					0.38	4	− 0.17	0.0048				
		Thre (mg)	0.19 (0.12–0.33)					0.13	2	−00.08	0.0013				
		Chol (mg)	61.16 (37.59–103.28)	50	5	−15.95	0.021					63	8	−2.95	0.63
	**Avg**^**b**^	Arg (mg)	0.23 (0.17–0.28					0.19	1	−0.04	0.0008				
		Chol (mg)	84.61 (62.76–103.47)	50	0	−34.55	< 0.0001					63	1	−21.54	< 0.0001
**O-MAINT**	**Min**^**a**^	CP (g)	9.19 (4.60–10.68)	3.97	0	−4.42	< 0.0001^*^					4.96	1	−3.43	< 0.0001^*^
		Arg (mg)	0.24 (0.12–0.28)					0.19	4	−0.03	0.05^*^				
		Phe + Tyr (mg)	0.74 (0.37–0.86)					0.38	1	−0.29	< 0.0001^*^				
		Chol (mg)	87.97 (44.05–102.32)	50	2	−30.33	0.0002^*^					63	3	−17.33	0.0022
	**Avg**^**b**^	Arg (mg)	0.24 (0.17–0.28)					0.19	1	−0.05	< 0.0001^*^				
**O-RESTRICT**	**Min**^**a**^	CP (g)	5.75 (4.94–6.35)	3.97	0	−1.75	< 0.0001					4.96	1	−0.76	< 0.0001
		Arg (mg)	0.15 (0.13–0.17)					0.19	16	0.04	< 0.0001				
		Chol (mg)	55.26 (47.36–60.80)	50	2	−4.82	0.0001					63	16	8.18	< 0.0001
	**Max**^**c**^	CP (g)	5.89 (4.94–6.58)	3.97	0	−1.90	< 0.0001					4.96	1	−0.91	< 0.0001
		Arg (mg)	0.15 (0.13–0.17)					0.19	16	0.04	< 0.0001				
		Chol (mg)	56.93 (47.36–63.06)	50	1	−6.25	< 0.0001					63	15	6.75	< 0.0001
	**Avg**^**b**^	CP (g)	5.83 (4.94–6.58)	3.97	0	−1.82	< 0.0001					4.96	1	−0.83	< 0.0001
		Arg (mg)	0.15 (0.13–0.17)					0.19	16	0.38	< 0.0001				
		Chol (mg)	55.98 (47.36–63.0)	50	1	−5.47	< 0.0001					63	15	7.53	< 0.0001

The daily intake of all essential nutrients was calculated from each cat’s food intake and the average nutrient content of the diet (Table 5) and expressed per kg BW ^0.67. Results are written as median (range)

*CP* Crude protein, *Arg* Arginine, *Phe + Tyr* Phenylalanine plus tyrosine, *Thre* Threonine, *Chol* Choline, *Min* Minimum, *Avg* Average, *Max* Maximum, *Req* Requirement, *No. Less* Number less, *Diff* Difference, *MR* Minimum requirement, *AI* Adequate intake, *RA* Recommended allowance

^*^Not normally distributed, expressed as signed-rank

^a^Minimum daily intake defined as the least daily intake that the cat consumed during the maintenance period

^b^Average daily intake was defined as the mean daily intake for the whole maintenance period

^c^Maximum daily intake defined as the greatest daily intake that the cat consumed during the maintenance period

^d^ All requirements are expressed as the unite stated per kg BW^0.67

^e^The number of cats with nutrient intakes less than the NRC cut-off based upon minimum, maximum and average daily intakes, respectively

^f^ Mean difference between intake and NRC requirement

^g^ Minimum requirement: the minimal concentration of a maximally bioavailable nutrient that will support a defined physiological state [4]

^h^Adequate intake: the concentration of nutrient demonstrated to support a defined physiological state when no minimal requirement has been demonstrated [4]

^i^ Recommended allowance: the concentration of nutrient demonstrated to support a defined physiological stage [4]

In the L-MAINT group, all cats had maximum and average intakes of crude protein that were above the NRC recommendations (Table 2). Four cats had minimum crude protein intakes below NRC RA and one of these cats was also below NRC MR (Table 3). The maximum intake of each amino acid was above NRC recommendations for all cats and most also had average and minimum intakes above NRC (Table 2). A number of cats had minimum intakes below NRC requirements for a few amino acids. Four cats had minimum intakes of phenylalanine + tyrosine and two cats had minimum intakes of threonine below the NRC AI (Table 3). Similar to the O-MAINT group, arginine was most affected. Nine cats in the L-MAINT group had a minimum intake of arginine that was less than the NRC AI and one of these cats also had an average intake below NRC AI (Table 3).

Although there were cats with intakes below NRC requirements for crude protein and a few amino acids, arginine was the only amino acid for which there was a positive mean difference between intake and requirement. A significant mean difference from AI was noted only for the O-RESTRICT group and not for the O-MAINT group, at minimum (mean difference 0.04; *P* ≤ 0.0001), maximum (mean difference 0.04; *P* ≤ 0.0001) and average intake (mean difference 0.38; *P* ≤ 0.0001) (Table 3). There was not a statistically significant mean difference from AI for the L-MAINT group (mean difference 0.01; *P* = 0.6) (Table 3).

### Comparison with NRC 2006 recommendations - vitamins

Dietary intake of all vitamins was greater than the NRC recommendations for all cats in all groups, except for choline (Table 4). When compared to MR for choline, 3 cats in the L-MAINT group were below this at minimum intake and 1 cat at average intake (Table 3). Two cats in the O-MAINT group had a minimum choline intake below MR (Table 3). Also, in the O-RESTRICT group, 2 cats did not reach MR for choline at minimum intake, 1 cat was below at maximum intake and 1 cat at average intake (Table 3). When compared to RA for choline, L-MAINT had 8 cats below this at minimum intake and 1 cat at average intake (Table 3). One cat in the O-MAINT group was below RA for choline at minimum intake (Table 3). All 16 cats in the O-RESTRICT group had a minimum choline intake below RA and 15 cats had a maximum and average intake also below RA (Table 3).

**Table 4:** Daily intake of essential vitamins in 14 lean cats during maintenance (L-MAINT) and in 16 obese cats during maintenance (O-MAINT) and during energy restriction (O-RESTRICT), compared to NRC minimum requirement, adequate intake and recommended allowance

Nutrient	NRC^g^			Dietary intake		
	NRC MR^d^	NRC AI^e^	NRC RA^f^	L-MAINT	O-MAINT	O-RESTRICT
**Minimum**^**a**^						
Choline (mg)	50	–	63	61.16 (37.59–103.28)	87.97 (44.05–102.32)	55.26 (47.36–60.80)
Cobalamin (ug)	–	0.44	–	2.27 (1.40–3.84)	3.27 (1.64–3.80)	2.05 (1.76–2.26)
Folate (ug)	0.015	–	0.019	0.14 (0.09–0.24)	0.20 (0.10–0.23)	0.13 (0.11–0.14)
Niacin (mg)	–	0.79	–	8.04 (4.94–13.57)	11.56 (5.79–13.45)	7.26 (6.22–7.99)
Pantothenic acid (mg)	0.11	–	0.14	0.84 (0.52–1.42)	1.21 (0.60–1.40)	0.76 (0.65–0.83)
Pyridoxine (mg)	0.05	–	0.06	0.61 (0.38–1.03)	0.88 (0.44–1.02)	0.55 (0.47–0.61)
Retinol (ug)	–	19.8	24.7	82.30 (50.58–138.98)	118.39 (59.28–137.69)	74.37 (63.73–81.83)
Riboflavin (mg)	–	0.079	0.099	0.44 (0.27–0.74)	0.63 (0.31–0.73)	0.39 (0.34–0.43)
Thiamine (mg)	–	0.11	0.14	0.96 (0.59–1.62)	1.38 (0.69–1.61)	0.87 (0.74–0.96)
Vitamin D3 (ug)	–	0.14	0.17	0.78 (0.48–1.32)	1.12 (0.56–1.31)	0.71 (0.60–0.78)
Vitamin E (mg)	–	0.74	0.94	18.87 (11.60–31.87)	27.15 (13.59–31.57)	17.05 (14.61–18.76)
**Maximum**^**b**^						
Choline (mg)	50	–	63	102.19 (85.79–105)	91.03 (81.58–102.32)	56.93 (47.36–63.06)
Cobalamin (ug)	–	0.44	–	3.80 (3.19–3.90)	3.38 (3.03–3.80)	2.11 (1.76–2.34)
Folate (ug)	0.015	–	0.019	0.23 (0.20–0.24)	0.21 (0.19–0.23)	0.13 (0.11–0.14)
Niacin (mg)	–	0.79	–	13.43 (11.28–13.8)	11.96 (10.72–13.45)	7.48 (6.22–8.29)
Pantothenic acid (mg)	0.11	–	0.14	1.40 (1.18–1.44)	1.25 (1.12–1.40)	0.78 (0.65–0.86)
Pyridoxine (mg)	0.05	–	0.06	1.02 (0.86–1.05)	0.91 (0.82–1.02)	0.57 (0.47–0.63)
Retinol (ug)	–	19.8	24.7	137.53 (115.45–141.3)	122.50 (109.78–137.69)	76.61 (63.73–84.86)
Riboflavin (mg)	–	0.079	0.099	0.73 (0.61–0.75)	0.65 (0.58–0.73)	0.41 (0.34–0.45)
Thiamine (mg)	–	0.11	0.14	1.61 (1.35–1.65)	1.43 (1.28–1.61)	0.89 (0.74–0.99)
Vitamin D3 (ug)	–	0.14	0.17	1.30 (1.10–1.34)	1.16 (1.04–1.31)	0.73 (0.60–0.80)
Vitamin E (mg)	–	0.74	0.94	31.53 (26.47–32.4)	28.09 (25.17–31.57)	17.57 (14.61–19.46)
**Average**^**c**^						
Choline (mg)	50	–	63	84.61 (62.76–103.47)	89.22 (63.24–102.32)	55.98 (47.36–63.0)
Cobalamin (ug)	–	0.44	–	3.14 (2.33–3.84)	3.31 (2.35–3.80)	2.08 (1.76–2.34)
Folate (ug)	0.015	–	0.019	0.19 (0.14–0.24)	0.20 (0.14–0.23)	0.13 (0.11–0.14)
Niacin (mg)	–	0.79	–	11.12 (8.25–13.60)	11.73 (8.31–13.45)	7.36 (6.22–8.28)
Pantothenic acid (mg)	0.11	–	0.14	1.16 (0.86–1.42)	1.22 (0.87–1.40)	0.77 (0.65–0.86)
Pyridoxine (mg)	0.05	–	0.06	0.85 (0.63–1.03)	0.89 (0.63–1.02)	0.56 (0.47–0.63)
Retinol (ug)	–	19.8	24.7	113.86 (84.45–139.25)	120.07 (85.10–137.69)	75.33 (63.73–84.78)
Riboflavin (mg)	–	0.079	0.099	0.60 (0.45–0.74)	0.64 (0.45–0.73)	0.40 (0.34–0.45)
Thiamine (mg)	–	0.11	0.14	1.33 (0.99–1.63)	1.40 (0.99–1.61)	0.88 (0.74–0.99)
Vitamin D3 (ug)	–	0.14	0.17	1.08 (0.80–1.32)	1.14 (0.81–1.31)	0.71 (0.60–0.80)
Vitamin E (mg)	–	0.74	0.94	26.11 (19.36–31.93)	27.53 (19.51–31.57)	17.27 (14.61–19.44)

The daily intake of all essential nutrients was calculated from each cat’s food intake and the average nutrient content of the diet (Table 5) and expressed per kg BW ^0.67. Results are written as median (range)

^a^Minimum daily intake defined as the least daily intake that the cat consumed during the maintenance period

^b^Maximum daily intake defined as the greatest daily intake that the cat consumed during the maintenance period

^c^Average daily intake was defined as the mean daily intake for the whole maintenance period

^d^ Minimum requirement: the minimal concentration of a maximally bioavailable nutrient that will support a defined physiological state [4]

^e^ Adequate intake: the concentration of nutrient demonstrated to support a defined physiological state when no minimal requirement has been demonstrated [4]

^f^ Recommended allowance: the concentration of nutrient demonstrated to support a defined physiological stage [4]

^g^ All requirements are expressed as the unite stated per kg BW^0.67

The only positive mean differences between intake and requirement with respect to choline were observed in the O-RESTRICT group and only for the difference from RA (Table 3) at minimum intake (mean difference 8.18; *P* ≤ 0.0001), maximum intake (mean difference 6.75; *P* ≤ 0.0001) and average intake (mean difference of 7.53; *P* ≤ 0.001).

## Discussion

Based on similar studies performed in dogs, it was expected that cats would have intakes of essential nutrients below NRC requirements with energy restriction for weight loss, even when fed a commercial therapeutic weight loss food. The findings of the current study showed that choline and arginine are of particular interest in obese cats undergoing energy restriction.

During energy restriction, all cats were below the NRC RA for choline at minimum intake and all except one were below RA at maximum and average intake. Moreover, two cats were below NRC MR at minimum intake and one cat also at maximum and average intake. Choline intake was not only below the NRC requirements during energy restriction; during the maintenance period there were lean and obese cats that were below NRC RA and NRC MR for choline, though only at minimum and average intakes. This was not surprising given similar findings in canine research [3, 5]. Choline is a vitamin like nutrient that has important bodily functions related to lipid metabolism. Firstly, it is part of phosphatidylcholine which makes up the membrane of very low-density lipoproteins, which are the vehicle for fat transport out of the liver [7]. Secondly, choline has a role as a methyl-donor which again is important for lipoprotein production but also for production of L-carnitine [8]. L-carnitine has a crucial role in fatty acid oxidation because it is necessary for entrance of fatty acids into the mitochondria – the site of oxidation in the cell. Obese cats already have an increased risk of hepatic lipidosis, which is characterized by fat accumulation in the liver and is the most common liver disease in cats [9]. This is due to a larger quantity of fatty acids that can be released from peripheral fat stores and pre-existing insulin resistance related to obesity [10, 11], which diminishes the insulin-induced inhibition of lipolysis [12]. The concern is that in obese cats energy restriction may induce fatty acid mobilization, but if choline is not present in enough quantity, there could be impaired transport of fat out of the liver as well as reduced fatty acid oxidation in the liver, leading to an accumulation of fat and subsequent hepatic lipidosis [9]. It is important to note that all cats in the present study remained healthy for the duration of the maintenance and energy restriction periods and no cat demonstrated any clinical signs of hepatic lipidosis. Weight loss was monitored closely to ensure cats were not losing weight too rapidly. Performing liver biopsies would have allowed for interpretation of liver fat content to assess for potential underlying, subclinical hepatic lipidosis, however, this was outside the scope of the study [9, 13].

Arginine is an indispensable amino acid and must be provided in the diet for cats [8]. In the present study, one lean and one obese cat fed at maintenance had an average arginine intake below NRC AI. However, during energy restriction, all obese cats had a minimum, average and even maximum intake of arginine less than the NRC AI. Arginine is involved in protein synthesis and is the key intermediate in the urea cycle [8]. Consequences of arginine deficiency can include ammonia intoxication due to a limitation of the urea cycle being able to function properly to excrete nitrogen as urea [14]. Ammonia intoxication has been demonstrated in the literature when young cats were fed an arginine-free diet [15]. The onset of clinical symptoms, including lethargy, emesis, vocalization, mouth frothing, hyperactivity, hyperesthesia, ataxia and extended limbs, occurred rapidly – within a few hours. It is important to note that all cats in the present study remained clinically healthy for the duration of the energy restriction period and no cat exhibited any clinical signs of arginine deficiency. It is also important to consider that in the study where ammonia intoxication has been reported, the diet fed was completely devoid of arginine, whereas in contrast, the diet fed to the cats in the present study was a complete and balanced diet which included arginine.

Intakes of nutrients in the present study were compared to NRC recommendations for adult maintenance. It is not known whether these requirements reflect the true nutrient requirements for obese cats or for obese cats being energy restricted. Adipose tissue used to be thought of as benign tissue that did not require any additional energy and so fat mass was not taken into consideration when determining a cat’s energy requirement. In recent years however, studies have demonstrated that in fact adipose tissue is metabolically active and could therefore require energy [16, 17]. This makes it challenging to estimate the true nutrient requirement of obese cats and raises questions regarding comparisons of requirements for maintenance of lean adult cats versus maintenance of obese adult cats. Furthermore, in order to achieve weight loss, calories must be restricted, but in doing so, intake of all nutrients will also be restricted. This is the rationale behind purpose formulated veterinary therapeutic weight loss foods – the calories are reduced but essential nutrient concentrations are enhanced, aiming at meeting recommendations for adult maintenance. What is not well understood is what the intake of those essential nutrients should be – are they required in the same amounts as for lean cats at maintenance or are there some nutrients that are required in less – or even greater – amounts. Given the uncertainty and lack of published requirements for obese cats fed at maintenance and obese cats undergoing energy restriction, the comparisons made in the present study were to the NRC requirements (MR, AI and RA) of cats at adult maintenance as those are currently available, but intakes of nutrients below these requirements need to be interpreted cautiously. There is a possibility that obese cats undergoing energy restriction could benefit from additional supplementation of some essential nutrients. As discussed above, choline has an important role in utilization of fat for energy and for transport of fat. It could be hypothesized that increasing the availability of dietary choline during energy restriction would enhance weight loss and reduce the risk of hepatic lipidosis. In humans, choline’s potential role in non-alcoholic fatty liver disease has been reviewed by Sherrif et al., 2016 and in one study, choline supplementation lead to a reversal of fat accumulation in the liver [18, 19]. Such studies in obese cats have not yet been performed and so this leaves room for future research in this area.

The consensus for treatment of obesity among veterinary professionals is to induce energy restriction [20, 21]. What is not agreed upon is the starting point for energy restriction. Various equations have been reported in literature [4, 5, 22–24], including the equation that was used in the present study [4, 22]. This equation is less conservative than other equations and provides energy allocation that is about 25% less than what is recommended by manufacturer of the food used for weight loss. One could argue that if a different equation was used, the obese cats would potentially have intakes of all essential nutrients within NRC requirements. While these theoretical calculations were not performed for the present study, it does leave room for future studies to investigate the effect of various levels of energy restriction on intake of essential nutrients. Still, in clinical practice, regardless of which energy equation is used, it is paramount in the successful weight loss plan to consider this as a starting point only and to evaluate success with frequent monitoring of the patient including measuring body weight and assessing BCS and MCS [25]. Safe weight loss rate for cats is recommended at 0.5 to 2% of starting body weight per week [4, 23] and caloric requirement should be adjusted based on whether or not this target is reached. Thus, an initial equation could be more conservative, but based on the cat’s response to this calorie amount, stricter restriction could be indicated. Furthermore, the degree of energy restriction reported in literature needed to induce hepatic lipidosis in cats is between 50 and 75% restriction of maintenance energy requirements [8, 26, 27]. In the present study, cats were fed 60% of maintenance energy requirements and were therefore not restricted to the same degree. Cats’ body weight, BCS and MCS were monitored. The average weight loss rate over the 10-week energy restriction period was 0.94 (+/− 0.28) % of initial body weight per week, well within the 0.5 to 2% range. Therefore, though the energy equation used may have been more restrictive than others, it appeared to be appropriate for cats to lose an adequate percent of body weight.

On average, cats lost an appropriate amount of BW, however, by the end of the 10-week period of energy restriction, no cat had reached its calculated ideal BW. In the present study, ideal BW was calculated based on BCS and current BW [21]. For obese cats with a BCS of 9/9, morphometry was used to determine body fat [28] and then ideal BW was calculated. While BCS and morphometric measurements have been validated against DEXA, the gold standard for body composition assessment [29–31], as acceptable methods for assessing body composition, there is room for human error with these methods. Performing DEXA before, during and after weight loss would have been ideal for not only determining percent body fat and calculating ideal BW, but also for measuring lean body mass and monitoring during the study period. This would have allowed for differentiation between a decrease in fat mass and a decrease in lean body mass.

The food used in the present study was a purpose formulated veterinary therapeutic weight loss food. All cats were transitioned onto this food for a period of 7 days before the study began. Owners were using gram scales to measure the food and were recording daily intake in a food log. Given that the owners were responsible for following instructions and recording accurately, using client owned cats for this study could be considered a limitation. It is possible that owners could have fed a food other than the weight loss food, fed treats, or measured or recorded imprecisely. However, the weight loss plan was successful during the restriction period based on the calculated weight loss rate and so it is thought that owners were compliant. Nevertheless, using client owned cats for this study was beneficial because it mimicked what a typical weight loss plan in a clinical setting could look like. Recommendations in a clinical setting are not standard. As discussed above, there is opportunity for variation when determining energy requirements. There is also a variety of cat foods available to owners of obese cats. For one, in contrast to dogs, fewer cat owners take their cats to a veterinarian [32] and so may be attempting a weight loss plan without veterinary assistance. A cat owner could decide to continue feeding a maintenance food but restrict the amounts of food fed. Alternatively, they may select an over the counter food at a pet store marketed as light or low calorie and think that they were selecting a weight loss food. Some veterinarians will also not recommend a therapeutic weight loss food at the start of a weight loss plan and may instead recommend portion control on the current diet or will base feeding recommendations off of label instructions [5, 21]. The present study only evaluated essential nutrient intake for one diet. Since intake of some essentials nutrients was below NRC recommendations on a purpose formulated weight loss food, it would be interesting to explore intakes of essential nutrients when foods not intended for weight loss are fed for energy restriction. Even though these foods are not meant to be restricted, because veterinarians and pet owners are restricting cats on these foods, it warrants investigation.

## Conclusion

The current study reports minimum, maximum and average daily intakes of essential nutrients in a population of client-owned lean and obese cats that were first fed to maintain body weight and then during a 10-week period of energy restriction for the obese cats. All cats remained healthy and there was no clinical evidence of a nutrient deficiency, however for some nutrients particularly arginine and choline, daily intakes were below the NRC requirements especially during energy restriction. The optimal nutrient intake during energy restriction is not known, however some nutrients of concern have been identified in the present study and warrant further investigation.

## Methods

### Experimental design

The study design has been described in detail in a previous publication [6]. Briefly, 30 client-owned cats were enrolled in the study and were fed a dry commercial therapeutic weight loss cat food for maintenance and weight loss (Table 5). Cats with a body condition score (BCS) or 4 to 5/9 were assigned to the lean group (*n* = 14, 10 males and 4 females) and cats with a BCS of 8 to 9/9 were assigned to the obese group (*n* = 16, 10 males and 6 females). All cats were spayed or neutered lived indoors and were between the ages of 2 and 7 years. Following a 1-week transition to the diet, both lean and obese cats were fed to maintain body weight (BW) for a period of 4 weeks. The eq. 100 kcal X kg BW^0.67^ [4], using current body weight, was used to determine the daily energy requirement of each cat in the lean group (L-MAINT). The energy requirement of each cat in the obese group (O-MAINT) was determined by the eq. 130 kcal X kg BW^0.4^ [4], using ideal BW. Ideal BW was calculated based on BCS and current BW [28]. For obese cats with a BCS of 9/9, morphometry was used to determine body fat [33] and then ideal BW was calculated. Owners were provided with gram scales that had been calibrated before distribution. Owners were given a demonstration on how to use the scale and were also instructed to weigh the food offered and any food remaining not eaten by the cat and recorded in a written food log. Using this data, daily food intake was determined. Following the 4-week weight maintenance period, the obese cats underwent energy restriction for weight loss for a period of 10 weeks. Energy requirement for weight loss was calculated using the eq. 0.6 X (130 kcal X kg BW^0.4) [4, 22] using ideal BW for each cat in the obese group (O-RESRICT). Bloodwork performed included an initial CBC and biochemistry to confirm health prior to enrollment, followed by a partial biochemistry panel, glucose, cholesterol and triglycerides (TG), after the 4-week maintenance period for L-MAINT and O-MAINT and again at the end of the 10 week restriction period for O-RESTRICT. Blood was collected from each cat via jugular or cephalic venipuncture and was centrifuged for 10 min at 3000 rpm after refrigeration at 4 °C for 2 h. Serum was removed and immediately submitted to the Animal Health Laboratory at the Ontario Veterinary College, Guelph, Canada and analyzed for glucose, cholesterol and triglycerides (TG) via photometry using a Roche Cobas 6000 c501 analyzer (Roche Diagnostics, Basel, Switzerland). The study protocol adhered to the University of Guelph Animal Use Protocol and was approved by the University of Guelph Animal Care Committee (#AUP 2496). Owners of the cats gave written informed consent for participation in the study.

**Table 5:** Analytical values of average nutrient content of the diet used for weight maintenance and energy restriction in the study cats

Nutrient	Weight loss diet
Kcal/kg Metabolizable Energy	4180
	Per 100 g as fed
***Proximate Analysis***	
*Moisture*	5.45
*Protein*	36.54
*Fat*	12.3
*Ash*	5.45
*NFE*	40.3
*Crude Fiber*	6.0
*Total Dietary Fiber*	17.45
***Amino Acids***	
*Arginine (g)*	0.961
*Histidine (g)*	0.656
*Isoleucine (g)*	1.223
*Methionine (g)*	2.817
*Methionine and Cysteine (g)*	3.89
*Leucine (g)*	3.413
*Lysine (g)*	1.513
*Phenylalanine (g)*	1.627
*Phenylalanine and Tyrosine (g)*	2.933
*Threonine (g)*	1.105
*Tryptophan (g)*	1.265
*Valine (g)*	1.57
*Taurine (g)*	0.436
***Vitamins***	
*Vitamin A (ug retinol)*	471
*Vitamin D3 (ug)*	4.47
*Vitamin E (mg)*	108
*Thiamine (mg)*	5.5
*Riboflavin (mg)*	2.5
*Pyridoxine (mg)*	3.5
*Niacin (mg)*	46
*Pantothenic acid (mg)*	4.8
*Cobalamin (ug)*	13
*Folate (ug)*	0.8
*Choline (mg)*	350

Commercial therapeutic weight loss food (dry), which contained chicken by-product meal, brewers rice, corn, gluten meal, powdered cellulose, dried tomato, pomace, flaxseed, dried beet pulp, chicken liver flavor, coconut oil, pork fat, lactic acid, potassium chloride, calcium sulfate, L-lysine, choline chloride, carrots, DL-methionine, vitamins (vitamin E supplement, L-ascorbyl-2-polyphosphate (source of vitamin C), niacin supplement, thiamine mononitrate, calcium pantothenate, pyridoxine hydrochloride, vitamin A supplement, riboflavin supplement, biotin, vitamin B12 supplement, folic acid, vitamin D3 supplement), taurine, L-carnitine, minerals (manganese sulfate, ferrous sulfate, zinc oxide, copper sulfate, calcium iodate, sodium selenite), mixed tocopherols for freshness, natural flavors, β-carotene

### Diet analyses

Cats in all groups (L-MAINT, O-MAINT and O-RESRICT) were fed the same diet throughout the duration of the study. The diet was a dry commercial therapeutic weight loss cat food formulated to provide all essential nutrients needed during weight loss and maintenance (Table 5). No other food or treats were allowed for the duration of the study. Diet analysis, including proximate analysis, amino acid and vitamin analysis were performed. Proximate analysis was performed at Bureau Veritas^1^. The food was analyzed for moisture by vacuum oven (AOAC 945.38), fat using acid hydrolysis (AOAC 922.06, 933.05), ash via combustion (AOAC 923.03), crude fibre (AOCS Ba 6a-05), and total dietary fibre (AOAC 991.43, 985.29). Nitrogen free extract (NFE) was calculated using 100-(%moisture + %fat + %protein + %ash), metabolizable energy was calculated using [(4 x %protein) + (9 x %fat) + (4 x %NFE)] [4], and protein was calculated from nitrogen using Kjeldahl method (N × 6.25) (AOAC 992.15). Amino acid analysis was performed at the University of Guelph Animal Biosciences Laboratory. Diet was analyzed in triplicate according to AOAC Official Method 994.12 Amino Acids in Feeds. The diet was analyzed for arginine, cystine, histidine, lysine, leucine, isoleucine, methionine, phenylalanine, threonine, tryptophan, tyrosine, valine and taurine using acid hydrolysis followed by ultra-performance liquid chromatography [34]. Vitamin analysis was performed at Bureau Veritas^1^ and included vitamin A by liquid chromatography (AOAC 992.04, 992.06), vitamin D by liquid chromatography (AOAC 982.29), vitamin E by gas chromatography (AOAC 992.03, 989.09), vitamin K by microbiological assay (AOAC 992.27), thiamin by fluorometry (AOAC 942.23), riboflavin by fluorometry (AOAC 970.65), pyridoxine by microbiological assay (AOAC 985.32 mod.), niacin by microbiological assay (AOAC 944.13), pantothenic acid by microbiological assay (AOAC 992.07), cobalamin by microbiological assay (AOAC 986.23), folic acid by microbiological assay (AOAC 2004.05), and choline by enzymatic acid hydrolysis (AOAC 999.14).

### Estimation of dietary nutrient intake

Crude protein, indispensable amino acid and vitamin intake was assessed in each of the three groups (L-MAINT, O-MAINT and O-RESTRICT). For each cat in each group, minimum, maximum and average daily intake of each nutrient was calculated based on the total grams of the food consumed and the nutrient concentrations from the analyses performed. Minimum daily intake was defined as the day when the least amount of food was consumed by the cat in either the 4-week (L-MAINT and O-MAINT) or 10-week period (O-RESTRICT) and the maximum daily intake was defined as day when the greatest amount of food was consumed by the cat in the same 4- or 10-week period. The average daily intake was defined as the mean daily intake for each cat during the entire 4- or 10-week period. These values were then compared to the NRC 2006 recommendations (per kg ideal body weight^0.67) including minimum requirement (MR), adequate intake (AI) and recommended allowance (RA).

### Statistical analysis

Statistical analysis was performed using SAS v 9.4^2^. Data was assessed for normal distribution using a Shapiro-Wilks test. Log transformation was applied when necessary to meet the assumptions of the ANOVA. A mixed procedure accounting for the random effect of cat was used to determine if there were differences between group for intake of each nutrient. A post-hoc Tukey-Kramer adjustment for multiple comparisons was made if the overall F test was significant. Proc univariate was used to compare between the intake of each nutrient and the requirement as per the NRC [4]. The differences were assessed for normality with a Shapiro-Wilk test. Wilcoxon sign rank test or student t-test were used to test the null hypothesis that the mean or median was equal to zero. A positive difference between mean intake of the nutrient and the requirement (MR, AI or RA) indicates an intake less than the requirement and a negative number an intake greater than the requirement. A difference of zero would indicate a mean intake identical to the requirement.

For blood parameters (glucose, cholesterol and TG), residuals were assessed for normal distribution using a Shapiro-Wilks test and a log transformation was performed on data that was not normal. For normally distributed data, a two-sided t-test was used to determine differences in concentration of each parameter between L-MAINT and O-MAINT and between L-MAINT and O-RESTRICT. A paired t-test was used to determine differences in parameter concentrations between O-MAINT and O-RESTRICT. For non-parametric data, a Wilcoxon Signed-Rank test was used. Data is expressed as mean ± SE for normally distributed data and as median (minimum-maximum) for non-parametric data. A *p*-value of less than 0.05 was considered significant.

## Data Availability

The datasets used and/or analysed during the current study are available from the corresponding author on reasonable request.

## Abbreviations

NRC: National Research Council
MR: Minimum requirement
AI: Adequate intake
RA: Recommended allowance
BCS: Body condition score
MCS: Muscle condition score
BW: Body weight
AOAC: Association of Official Analytical Chemists
NFE: Nitrogen free extract
DEXA: Dual-energy x-ray absorptiometry
TG: Triglycerides

## Footnotes

1 Bureau Veritas Laboratories, Mississauga, Ontario, Canada

2 SAS Campus Drive, Cary, North Carolina, USA
